# Glacier-Fed Stream Biofilms Harbor Diverse Resistomes and Biosynthetic Gene Clusters

**DOI:** 10.1128/spectrum.04069-22

**Published:** 2023-01-23

**Authors:** Susheel Bhanu Busi, Laura de Nies, Paraskevi Pramateftaki, Massimo Bourquin, Tyler J. Kohler, Leïla Ezzat, Stilianos Fodelianakis, Grégoire Michoud, Hannes Peter, Michail Styllas, Matteo Tolosano, Vincent De Staercke, Martina Schön, Valentina Galata, Paul Wilmes, Tom Battin

**Affiliations:** a Systems Ecology Group, Luxembourg Centre for Systems Biomedicine, University of Luxembourg, Esch-sur-Alzette, Luxembourg; b River Ecosystems Laboratory, Alpine and Polar Environmental Research Center, Ecole Polytechnique Fédérale de Lausanne, Lausanne, Switzerland; University of Minnesota Twin Cities

**Keywords:** glacier-fed streams, metagenomics, antimicrobial resistance, biosynthetic gene clusters, cross-domain interactions, freshwater ecosystems

## Abstract

Antimicrobial resistance (AMR) is a universal phenomenon the origins of which lay in natural ecological interactions such as competition within niches, within and between micro- to higher-order organisms. To study these phenomena, it is crucial to examine the origins of AMR in pristine environments, i.e., limited anthropogenic influences. In this context, epilithic biofilms residing in glacier-fed streams (GFSs) are an excellent model system to study diverse, intra- and inter-domain, ecological crosstalk. We assessed the resistomes of epilithic biofilms from GFSs across the Southern Alps (New Zealand) and the Caucasus (Russia) and observed that both bacteria and eukaryotes encoded twenty-nine distinct AMR categories. Of these, beta-lactam, aminoglycoside, and multidrug resistance were both abundant and taxonomically distributed in most of the bacterial and eukaryotic phyla. AMR-encoding phyla included Bacteroidota and Proteobacteria among the bacteria, alongside Ochrophyta (algae) among the eukaryotes. Additionally, biosynthetic gene clusters (BGCs) involved in the production of antibacterial compounds were identified across all phyla in the epilithic biofilms. Furthermore, we found that several bacterial genera (*Flavobacterium*, *Polaromonas,* Superphylum Patescibacteria) encode both atimicrobial resistance genes (ARGs) and BGCs within close proximity of each other, demonstrating their capacity to simultaneously influence and compete within the microbial community. Our findings help unravel how naturally occurring BGCs and AMR contribute to the epilithic biofilms mode of life in GFSs. Additionally, we report that eukaryotes may serve as AMR reservoirs owing to their potential for encoding ARGs. Importantly, these observations may be generalizable and potentially extended to other environments that may be more or less impacted by human activity.

**IMPORTANCE** Antimicrobial resistance is an omnipresent phenomenon in the anthropogenically influenced ecosystems. However, its role in shaping microbial community dynamics in pristine environments is relatively unknown. Using metagenomics, we report the presence of antimicrobial resistance genes and their associated pathways in epilithic biofilms within glacier-fed streams. Importantly, we observe biosynthetic gene clusters associated with antimicrobial resistance in both pro- and eukaryotes in these biofilms. Understanding the role of resistance in the context of this pristine environment and complex biodiversity may shed light on previously uncharacterized mechanisms of cross-domain interactions.

## INTRODUCTION

Today, antimicrobial resistance (AMR) has become a well-known threat to human health with an estimated number of 700,000 people per year dying of drug-resistant infections (1). The dramatic rise of antimicrobial resistance over the past decade has even led to the moniker “silent pandemic” (2). Therefore, AMR is often directly associated with human impacted environments with a global increase in resistant bacteria linked to the over- and misuse of antibiotics (3). However, contrary to public perception, AMR is a natural phenomenon, which has existed for billions of years (4). Long before the rather recent use of antibiotics in the clinical setting, microorganisms have used these, along with corresponding protective mechanisms, to establish competitive advantages over other microbes contending for the same environment and/or resources (5).

Microorganisms, in general, produce a range of secondary metabolites with diverse chemical structures, which in turn confer a variety of functions, including antibiotics (6). Such secondary metabolites, including metal transporters and quorum-sensing molecules (7, 8) are associated with enhanced fitness by driving growth, including by inhibiting the growth of other bacteria by depleting iron through increased capacity to compete for this essential resource. Consequently, many of these natural products have found their applications in industrial settings, but also as anti-infective drugs in human medicine (7, 9, 10). The biosynthetic pathways responsible for producing these specialized metabolites are encoded by locally clustered groups of genes known as “biosynthetic gene clusters” (BGCs). Typically, BGCs include genes for expression control, self-resistance, and metabolite export (11). They can, however, be further divided into various classes, including nonribosomal peptide synthetases (NRPSs), type I and type II polyketide synthases (PKSs), terpenes, and bacteriocins, alongside others (10). NRPSs and PKSs specifically have been of interest due to their known synthesis of putative antibiotics (12, 13). Furthermore, evidence suggests that within these BGCs at least one resistance gene conferring resistance can be found as a self-defense mechanism against the potentially harmful secondary metabolites encoded by the BGC (14). For instance, the tylosin-biosynthetic gene cluster of Streptomyces fradiae also encodes three resistance genes (*tlrB*, *tlrC*, and *tlrD*) (15), while in another report, *Streptomyces toyacaensis*, the *vanHAX* resistance cassette, is proximal to the vancomycin biosynthesis gene cluster, thereby encoding inherent resistance (16).

Remote and pristine microbial communities provide a potentially rich genetic resource to explore naturally occurring antibiotic resistance from the preantibiotic era. These phenomena may currently only be evident in a few environments with limited anthropogenic influence (e.g., permafrost, glaciers, deep sea, and polar regions). These ARGs and resistant bacteria evolving in pristine environments may therefore be considered the inherent antibiotic resistance present in the environment (5). Despite this, some recent reports suggest that even these remote microbial communities may be coming under the influence of anthropogenic antimicrobial resistance (17), thus necessitating the need to characterize these relatively pristine environments before their imminent decline due to climate change.

We have recently reported the genomic and metabolic adaptations of epilithic biofilms to environmental conditions in glacier-fed streams (GFSs) (18). For example, given the short flow season during glacial melt in summer, the incentive to reproduce quickly while conditions are favorable, is high. During these so-called windows of opportunity, the necessity for taxa to appropriate resources, especially when they are limiting as in GFSs, yields a competitive environment. Within these biofilms, we observed cross-domain interactions and internal recycling of nutrients between microorganisms to potentially mitigate the harsh nutrient and environmental conditions of the GFSs. Additionally, owing to complex biodiversity (19) and generally oligotrophic conditions (20) in GFSs, epilithic biofilms are ideal model systems for understanding BGCs and AMR in the context of cooccurring microorganisms. Our previous insights revealed that taxa such as *Polaromonas*, *Acidobacteria*, and *Methylotenera* have strong interactions with eukaryotes such as algae and fungi (21). The inherent diversity allows for understanding the influence of AMR in microbial interactions as evidenced by bacterial–fungal interactions leading to the discovery of penicillin by Alexander Fleming in 1928 (22). Along similar lines, Netzker et al. (23) reported that microbial interactions lead to the production of bioactive compounds, including antibiotics potentially shaping the microbial consortia within a community.

Here, to shed light on the role of AMR in shaping microbial communities within pristine environments, we used whole-genome shotgun sequencing (metagenomics) to investigate twenty-one epilithic biofilms from glacier-fed streams. These samples were collected from 8 GFSs spread across the Southern Alps in New Zealand and the Caucasus in Russia (Table S1). Herein, we found 29 categories of ARGs within the GFSs across both bacterial and eukaryotic domains. Importantly, most of the AMR was found in bacteria. We also identified antibacterial BGCs that were encoded both in bacterial and eukaryotic metagenome-assembled genomes (MAGs). Our findings demonstrate that microorganisms within biofilms from pristine environments not only encode ARGs, but that they may potentially influence several features of epilithic biofilms such as biofilm formation, community assembly, and/or maintenance, including conferring potential mechanisms for competitive advantages under extreme conditions.

## RESULTS

### Antimicrobial resistance in a pristine environment.

**FIG 1:**
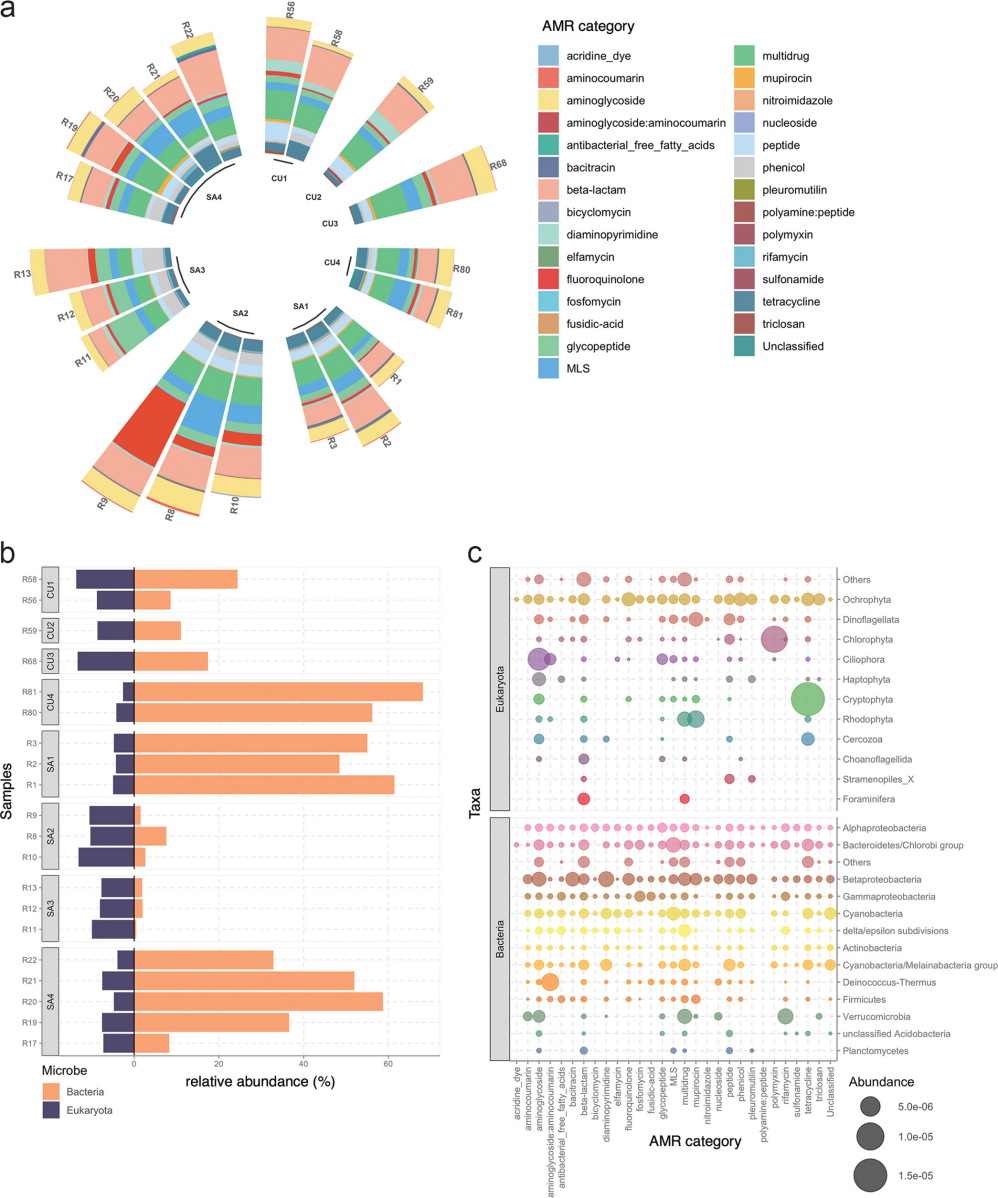
Epilithic biofilms in GFSs harbor a diverse resistome. (a) Relative abundance of 29 AMR categories within 21 epilithic biofilms collected from four New Zealand Southern Alps (SA) and four Russian Caucasus (CU) GFSs. (b) Bar plots depicting the relative abundance of bacteria and eukaryotes encoding ARGs. (c) Phylum-level representation of the AMR abundances across bacteria and eukaryotes. Size of the closed circle indicates the normalized relative abundance (RNum_Gi; see Materials and Methods), and the color represents individual phyla.

We characterized the resistomes of GFS epilithic biofilms and assessed the distribution of AMR in twenty-one epilithic biofilm samples, across 8 individual glaciers originating from the Southern Alps in New-Zealand (SA1, SA2, SA3, and SA4) and the Caucasus in Russia (CU1, CU2, CU3, CU4). In total, we identified a high number (*n* = 1,840) of ARGs within 29 categories of AMR, with similar AMR profiles observed across all GFSs (Fig. 1a), except for CU1, CU2, and SA3, where the differences were driven by elevated fluoroquinolone, peptide, glycopeptide, and phenicol resistance (Fig. S1). It is to be noted that while ARGs refer to the genes encoding specific resistance, AMR categories derived from metagenomic data in this context, typically reflect the functional potential associated with respect to the resistance encoded. Of the identified AMR categories, beta-lactam and multidrug resistance (i.e., resistance conferring protection against multiple antibiotic classes), followed by aminoglycoside resistance, were found to be highly abundant in all samples. We subsequently analyzed the diversity of ARGs within the various resistance categories and found beta-lactam resistance to represent the largest resistance category, contributing 930 unique ARGs to the resistome. This was followed by multidrug (179 ARGs) and aminoglycoside (176 ARGs) resistance (Table S2). In contrast, some resistance categories such as polymyxin and pleuromutilin resistance were only detected at very low levels within the epilithic biofilm resistomes.

We further investigated the contribution of microbial populations to the resistome and found contributions from both prokaryotes and eukaryotes (Fig. 1b). Prokaryotes within this study refer to bacteria alone, since archaea encoded an infinitesimal number of ARGs (<0.000001% RNum_GI; see Materials and Methods.), and therefore were excluded from further analyses. Among the eukaryotes, the phylum Ochrophyta (algae) was the dominant contributor and encoded most of the AMR categories (Fig. 1c; Fig. S2a). In bacteria, AMR was more evenly distributed, with most of the phyla encoding ARGs across all categories (Fig. 1c). However, members of the Alphaproteobacteria, Betaproteobacteria, and the Bacteroidetes/Chlorobi group encoded the highest overall ARG abundance (Fig. 1c; Fig. S2b). Additionally, AMR categories such as aminoglycoside, beta-lactam, glycopeptide, and rifamycin resistance (among others) were widely distributed in both bacteria as well as among the eukaryotes. On the other hand, categories such as aminocoumarin, bacitracin, and diaminopyrimidine resistance were found to be primarily encoded by bacteria.

**FIG 2:**
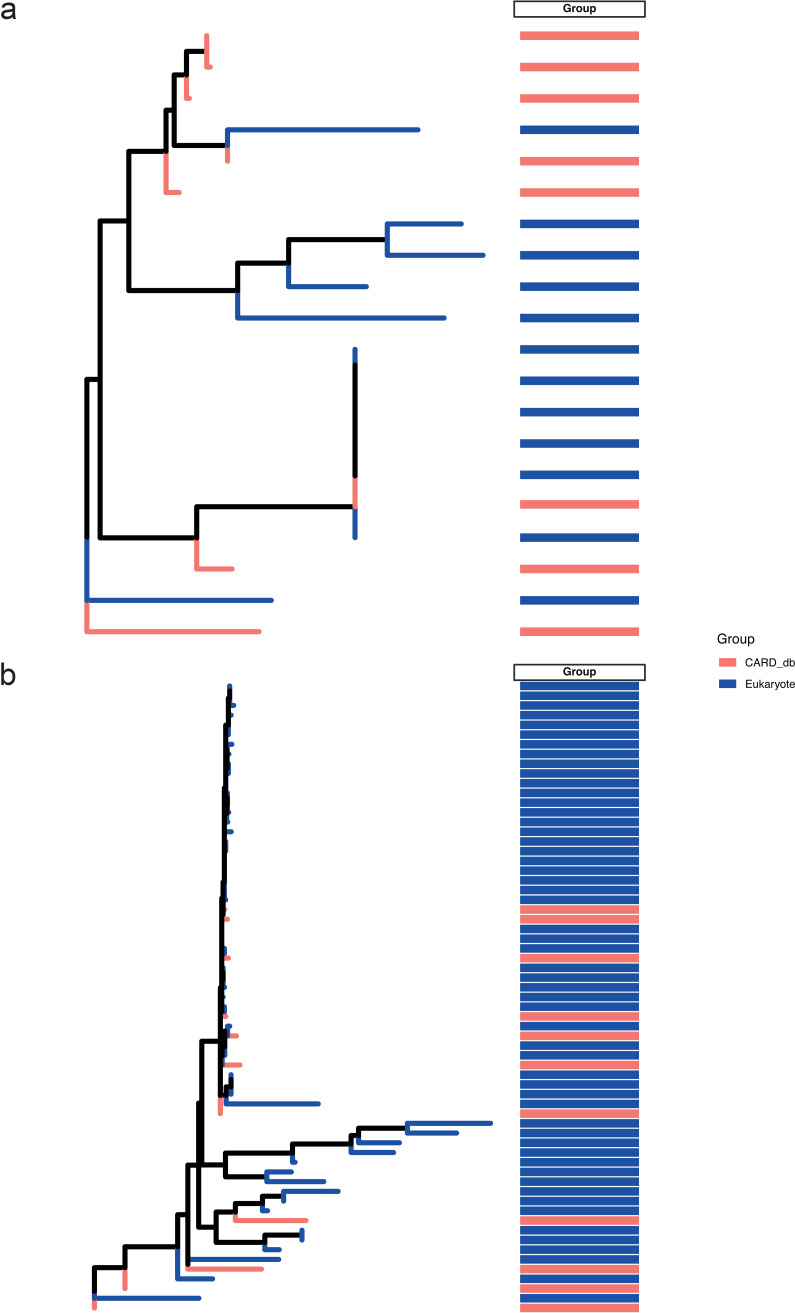
Phylogenetic analyses of putative eukaryotic ARGs. (a) Phylogenetic tree depicting the relatedness and potential homology between ARG sequences from the reference database, i.e., CARD, with the TETA (tetracycline) resistance gene sequence retrieved from GFS eukaryotes. (b) Tree built using the ERM (MLS phenotype) resistance genes retrieved from the eukaryotes and CARD databases. Group in both figures indicates the origins of the sequence, i.e., CARD database or eukaryote.

Given the novelty of the identification of putative ARGs in eukaryotes, we applied additional checks to validate the ARG sequences. First, the eukaryotic ARG sequences were assessed for homology with known bacterial representatives from the CARD database. For example, both TETA (tetracycline) and ERM (MLS phenotype) sequences were the most representative for ARGs in ~95% of the eukaryotic MAGs and were therefore retrieved from the eukaryotic contigs. Additionally, these genes were chosen for assessment given the availability of different isoforms in CARD. Phylogenetic analyses subsequently revealed that several of the eukaryotic-encoded ARGs indeed shared considerable homology with their bacterial counterparts (Fig. 2), as identified by their grouping within clades. A similar phenomenon was observed when comparing the ARGs retrieved from the GFS bacteria with the CARD reference ARGs (Fig. S3). Interestingly, several eukaryotic ARGs were distantly related, raising the hypothesis that they may be naturally occurring enzymes that may be distantly related homologs of the reference ARGs. To account for this possibility, all the ARG sequences from the eukaryotes were compared against the UniProtKB database (Fig. S4a and Table S3). With both TETA and ERM, we found that a majority (>73%) of the prokaryotic ARGs had hits in the database with at least 50% identity covering 90% of the total sequence length. Only ~8% of the eukaryotic ARGs demonstrated 50% identity with the database sequences, albeit with high E values suggesting poor or partial homology with existing reference sequences. We manually verified the partially homologous sequences from eukaryotic TETA resistance genes and found weak homology (<58%) to the existing list of entries in UniProtKB (representative example: Fig. S4b). Among the amino acids with substantial homology in terms of their positions, we found that 141 to 159 aa from the TETA genes found in UniProtKB were partially homologous with those of the eukaryotes’ TETA genes. Nevertheless, it is at present unclear whether these amino acids form the active sites conferring tetracycline resistance. Due to the lack of functional information, we additionally used fARGene (24) to determine the likelihood that functional resistance genes were encoded by these genes and to subsequently classify any novel antibiotic resistance genes. Although fARGene classified and identified all bacterial ARGs, eukaryote-encoded ARGs were unclassified, likely due to their novelty or the lack of appropriate representatives in the database underlying fARGene.

### Antibiotic biosynthesis pathways and biosynthetic gene clusters.

As described above, beta-lactam, multidrug, and aminoglycoside resistance were the most abundant resistance categories within GFS epilithic biofilms. This was not surprising as beta-lactams and aminoglycosides are natural and prevalent compounds (25, 26). Furthermore, multidrug resistance is typically conferred via efflux machineries which were also common in the GFS epilithic biofilms. These typically serve dual purposes in particular for protein export within most bacteria (27). Based on these results, it is therefore likely that pristine environments such as GFSs potentially reflect the spectrum of natural antibiotics and their resistance mechanisms, reinforcing their capacity to serve as natural baselines for assessing enrichments and spread of AMR.

To further understand if these encoded resistance genes reflected natural antibiotic pressure, we investigated pathways associated with antibiotic biosynthesis using the KEGG (Kyoto Encyclopedia of Genes and Genomes) database (28). In total, we identified seven different pathways corresponding to the biosynthesis of macrolides (MLS), ansamycins, glycopeptides (vancomycin), beta-lactams (monobactam, penicillin, and cephalosporin), aminoglycosides (streptomycin), and tetracyclines, which were present in various abundances in all samples (Fig. S5a). Importantly, the identified antibiotic synthesis genes thereby corresponded to the resistance categories identified within the epilithic biofilms. Interestingly, in most of the GFSs, antibiotic biosynthesis was primarily encoded by bacteria spanning multiple phyla (Fig. S5b, c). Exceptions to these were GL11 and GL15 in which biosynthesis pathways were equally distributed among eukaryotes, specifically Ochrophyta, in addition to bacteria.

**FIG 3:**
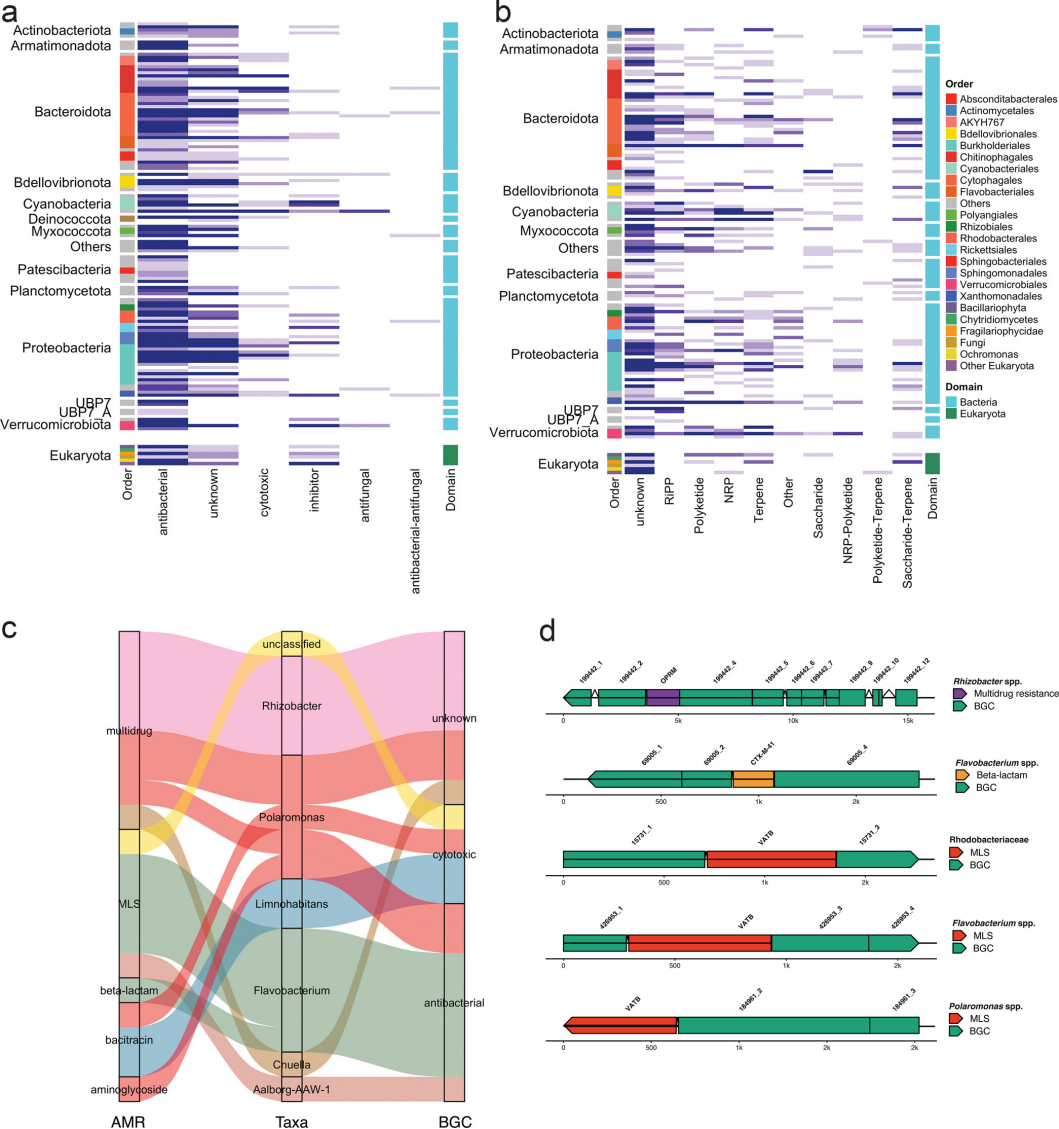
Biosynthetic gene clusters indicate the resistome potential. (a) Heatmap depicting the overall abundance of BGCs identified across bacterial and eukaryotic MAGs. The respective phyla are listed on the left while the colored legend represents the taxonomic order. (b) In-depth characterization of the “antibacterial” BGCs found within all phyla and orders across medium-to-high quality MAGs. (c) Alluvial plots depicting the taxa where both BGCs and AMR were found adjacently on the same contig. Colours indicate the genera associated with the MAGs. (d) Genome plots indicating the location of the ARGs in relation to the biosynthetic gene clusters.

KEGG pathways described for the production of certain antibiotics tend to contain enzymes involved in far-upstream reactions. For example, the “streptomycin biosynthesis” pathway in KEGG ortholog (ko00521) is defined with 21 KOs, including general and widespread genes such as glucose metabolism. Therefore, to further validate our observations regarding the antibiotic biosynthesis pathways, we assessed the abundance of BGCs, in the epilithic bioficlms within the MAGs, that are known to encode genes for secondary metabolite synthesis, including antibiotics. We found six different structural classes of BGCs by annotating 537 medium-to-high-quality (>50% completion and <10% contamination) bacterial and 30 eukaryotic MAGs using antiSmash (29) and DeepBGC (30). Using this ensemble approach, we identified one or more BGCs in most bacterial (*n* = 490, ~91% of all bacterial MAGs) and eukaryotic (*n* = 28) MAGs. Of these BGCs, those annotated with an antibacterial function were dominant across the microbial populations, represented here by the MAGs, and were found across all phyla (Fig. 3a). Overall, a wider variety of BGCs associated with cytotoxic activity, inhibitory, and antifungal mechanisms were also identified in bacteria. Eukaryotes, on the other hand, encoded a high prevalence of antibacterial BGCs (~93% of all eukaryotic MAGs) (Fig. 3a). We further annotated those BGCs identified as antibacterial to determine their subtypes and found that most of them were “unknown” (Fig. 3b). However, other identified subtypes include ribosomally synthesized and posttranslationally modified peptides (RiPPs) such as bacteriocins, along with NRPs, PKs, and terpenes. In line with the abundance of aminoglycoside and beta-lactamase resistance genes, we found that ~19 of the MAGs encoded beta-lactamase gene clusters, while ~15% encoded aminoglycoside-associated BGCs, followed by BGCs of “unknown” classification.

According to the resistance hypothesis (14), within or close to each BGC there is at least one gene conferring resistance to its encoded secondary metabolite. To test this, we assessed whether the MAGs encoding a BGC also encoded corresponding ARGs. In line with this hypothesis, we identified BGCs and their respective resistance genes in close proximity to each other through their localization on the same contig. Consequently, we identified various BGCs encoded together with ARGs in both the bacterial and eukaryotic MAGs. For example, we found that an antibacterial BGC was encoded by *Flavobacterium* spp. on the same contig as both MLS (i.e., macrolides, lincosamides, and streptogramin) and beta-lactam resistance genes (Fig. 3c). Incidentally, we also found that a candidate phyla radiation (CPR) bacterium (Aalborg-AAW-1; phylum Patescibacteria) also encoded both antibacterial BGC and MLS resistance on the same contig. Further closer inspection revealed that the ARGs were encoded within the BGCs in certain cases, while in other instances the ARGs directly flanked the BGC itself (Fig. 3d). Importantly, we found that the closely encoded ARGs and BGCs were localized within a distance of 100 bp to 2 Kb of each other.

## DISCUSSION

Microbial reservoirs in pristine environments, with little to no impact from anthropogenic selection pressures, provide the opportunity to investigate the natural propensity and linked evolutionary origins of AMR. Here, by leveraging metagenomics on twenty-one epilithic biofilms, we assessed the resistomes of eight individual GFS epilithic biofilms.

To date, while many studies have looked for novel antibiotics and resistance genes in pristine environments such as the deep sea (31) or the polar regions (32), few have explored the full diversity of antibiotic resistance in such environments (33, 34). Van Goethem et al. (35) identified 117 naturally occurring ARGs associated with multidrug, aminoglycosidem and beta-lactam resistance in pristine Antarctic soils (17). Similarly, D’Costa et al. (4) identified a collection of ARGs encoding resistance to beta-lactams as well as tetracyclines and glycopeptides in 30,000-year-old Beringian permafrost sediments. In agreement with these previous studies, we identified 29 AMR categories, including the previously mentioned resistance categories, in the studied biofilm communities. Among these, the highest ARG abundance was associated with aminoglycoside and beta-lactam resistance. Our study further suggests that although the overall abundance differs, the epilithic resistome was highly similar in all GFSs, independent of origin (i.e., New Zealand or Russia). Furthermore, our results agree with the results obtained in other resistomes identified in pristine environments such as Antarctic soils and permafrost in terms of the identified ARGs. Unlike previous studies, where ARGs were primarily associated with bacteria, we report for the first time that AMR was associated with both bacteria and eukaryotes in various abundances in environmental samples, including GFSs. A previous study by Brown et al. (36) reported that the IRS-HR (isoleucyl-tRNA synthetase—high resistance) type gene conferring resistance against mupirocin was identified in Staphylococcus aureus. More importantly, they suggested that horizontal gene transfer led to the acquisition of IRS-HR genes by bacteria from eukaryotes (36). Despite these early reports, the contribution of eukaryotes to most resistomes, including from pristine environments, has largely been unexplored thus far. An exception to this was the report by Fairlamb et al. (37), who identified eukaryotic drug resistance, especially encoded by fungi (*Candida* and Aspergillus) and parasites (*Plasmodium* and *Trypanosoma*). However, most of these modes of resistance were highly specific toward particular drug treatments (37). Our results specifically revealed that taxa from the phylum Ochrophyta encoded resistance to 28 AMR categories, and this was also reflected in other (micro-)eukaryotes. Interestingly, phylogenetic analyses revealed shared homology between the ARGs from the GFSs compared to reference databases. There were also other ARGs that formed separate clades indicating that they may either be distantly related or previously unidentified resistance genes. While going beyond the scope of the present study, the distantly related ARGs would need to be experimentally validated as performed in previous studies on phage-borne ARGs (38) and novel ARGs (24). Importantly, dinoflagellates are known to produce biologically active natural compounds that have been implicated in ecological roles and thus have been targeted in bioprospecting drives (39). However, although the anti-inflammatory (40) and vasoconstrictive (41) activities of dinoflagellate-derived biomolecules have been well-defined, dinoflagellate-encoded BGCs have been largely unknown. It must be noted that our analyses provide insights on the functional potential of the identified ARGs, within both pro- and eukaryotes. Substantial additional wet-lab experiments are required to validate the function of the identified ARGs, especially within the identified eukaryotic genomes.

Apart from encoded resistance mechanisms, microalgae such as Ochrophyta have been of interest as a source of (new) antimicrobial compounds (42, 43). Martins et al. reported that extracts from different microalgae may potentially serve not only as antimicrobial agents, but also as anticancer therapeutics. However, our present results suggest that these taxa may also serve as environmental reservoirs for AMR itself. It is however presently unclear whether this phenomenon confers advantages with respect to niche occupation and protection against bacterial infection as well as whether the eukaryotes are sensitive to the antibiotics produced by them. Our attempts at trying to elucidate putative or potential functions using tools such as fARGene and through databases such as UniProtKB were unsuccessful. Although we found partial homology of the eukaryote-encoded TETA genes to those found in UniProtKB (141 to 159 aa), it is unclear whether the homologous regions represent an active site and/or the expression of the ARG. This circumstance further highlights the lack of sufficient knowledge highlighting the need for additional wet-lab validations of our findings.

Studies delving into the origins of AMR have reported that fecal pollution may explain ARG abundances in anthropogenically impacted environments (44). This phenomenon was also observed by Antelo et al. (45) and others (46) who detected ARGs in soils in Antarctica, especially in proximity to scientific bases. A similar trend manifested in our analyses, where quinolone (synthetic antibiotic) resistance was found in the glacier streams from the Southern Alps. Interestingly, we observed that the quinolone resistance was higher in a glacier that was accessible to hikers along the Cascade Saddle, a famous trekking hot spot. This observation is along the lines of a recent report by Hwengwere et al. suggesting that the concentrations of anthropogenically derived ARGs are greater in regions closer to the research stations in Antarctica (17). Although it is plausible that some of the GFSs sampled in our study may indeed be under anthropogenic influence, albeit minimal, the majority of them are considered to be pristine environments. In this context, AMR is likely derived from natural antibiotics produced by microorganisms as a competitive advantage given the oligotrophic conditions. Microorganisms acquire resistance either as a protective measure against other microorganisms (47, 48) or as a self-defense mechanism to prevent inadvertent suicide by damaging metabolites (14). Accordingly, we found both antibiotic biosynthesis pathways and BGCs within the epilithic resistomes. We identified pathways for the biosynthesis of glycopeptides, beta-lactams, and aminoglycosides, among others, concurrent with the high abundance of ARGs against said antibiotics. Additionally, we identified BGCs with a predicted antibacterial function in both eukaryotes and bacteria. While a limited number of studies such, as Waschulin et al. (49) and Liao et al. (50), have shown BGCs in pristine environments, none of these studies have contextualized the cooccurrence of BGCs with AMR. Hence, we not only found that most of our MAGs contain BGCs, of which many have an antibacterial function, but also found all MAGs to encode multiple resistance genes. Additionally, we found several BGCs closely localized to ARGs on the same contig, thereby indicating an immediate self-defense mechanism against the encoded secondary metabolites. This agrees with the resistance hypothesis highlighted by Tran et al. stating that a gene conferring resistance to potentially harmful metabolites produced by the organism is to be found within the BGC-encoding operons (14). We also observed that the recently identified CPR bacteria (51) (in our case, phylum Patescibacteria) not only encoded AMR but also harbored genes associated with the production of molecules with antibacterial effects. Although Patescibacteria have been identified in oligotrophic environments (52, 53) with carbon and/or nutrient limitations similar to those observed for GFSs, it is plausible that their ability to survive with minimal biosynthetic and metabolic pathways may indeed depend on the expression of BGCs and AMR. At the time of this writing, a preprint by Maatouk et al. (54), described the presence of ARGs across publicly available CPR bacterial genomes. In addition, we report the identification of AMR within GFS-derived CPR genomes, likely as a means of competitive inhibition against other taxa. Alternatively, biofilms may also allow for collective resistance, tolerance, and exposure protection to antibacterial compounds (55). The AMR and BGCs encoded by most phyla may therefore affect cooperation and/or interactions associated with nutrient exchange, leading to the privatization of public goods (55). Such a phenomenon may be achieved due to the competition within taxa, both at the intra- and interspecies levels, via secretion of toxins (48) and occupying spatial niches (56, 57) thereafter. Furthermore, Stubbendieck and Straight previously highlighted the multifaceted effects of bacterial competition which include the potential taxation and subsequent increase in bacterial fitness (58). Thus, the *in-situ* competition within multispecies biofilms may allow for cross-phyla and cross-domain interactions while simultaneously increasing the overall fitness of the endogenous epilithic microbial community. Alternatively, these interactions or lack thereof may shape the overall community, including spatial organization (59), especially in energy-limited systems such as the GFSs.

### Conclusions.

Epilithic biofilms are an integral and key mode of survival in the extreme environment of glacier-fed streams. Herein, we report that these biofilms provide critical insights into the naturally occurring resistome. Our findings shed light on the survival mechanisms via the resistome, including potential ecological dimensions of ARGs and BGCs within the GFS microbial communities. Furthermore, we reveal the congruence of genes encoding both BGCs and AMR, in both bacteria and eukaryotes. More importantly, we highlight for the first time the comprehensive AMR profile of CPR bacteria and of (micro-)eukaryotes. Collectively, our results highlight underlying resistance mechanisms, including BGCs, employed in “biological warfare” in oligotrophic and challenging glacier-fed stream ecosystems.

## MATERIALS AND METHODS

### Sampling and biomolecular extractions.

Eight GFSs were sampled in early to mid-2019 from the New Zealand Southern Alps and the Russian Caucasus, respectively, for a total of 21 epilithic biofilms (Table S1). The biofilm samples were collected from each stream reach, depending on the stream geomorphology, i.e., the presence of boulders, as described previously by Busi et al. (18). One to three biofilm samples were collected per reach (Table S1), taken using sterilized metal spatulas to scrape rocks, followed by their immediate transfer to cryovials. Samples were immediately flash-frozen in liquid nitrogen and stored at −80°C until DNA was extracted. DNA from the epilithic biofilms was extracted using a previously established protocol (60) adapted to a smaller scale due to relatively high DNA concentrations. DNA quantification was performed for all samples with the Qubit dsDNA HS kit (Invitrogen).

### Sequencing and data processing for metagenomics.

Random shotgun sequencing was performed on all epilithic biofilm DNA samples after library preparation using the NEBNext Ultra II FS library kit. Fifty nanograms of DNA was enzymatically fragmented for 12.5 min, and libraries were prepared with six PCR amplification cycles. An average insert of 450 bp was maintained for all libraries. Qubit was used to quantify the libraries followed by sequencing at the Functional Genomics Centre Zurich on a NovaSeq (Illumina) using a S4 flowcell. The metagenomic data were processed using the Integrated Meta-omic Pipeline (IMP v3.0; commit# 9672c874 available at https://git-r3lab.uni.lu/IMP/imp3) (61). IMP’s workflow includes preprocessing, contig assembly, genome reconstruction (metagenome-assembled genomes, i.e., MAGs), and taxonomic and additional functional analysis of genes based on custom databases in a reproducible manner (61). Eukaryotic binning was performed in addition to the processing with the IMP workflow as described in Busi et al. (18). Briefly, eukaryotic MAGs were binned using CONCOCT (62), using contigs that were identified as eukaryotic, including those not mapping to bacteria or viruses in the maxikraken2 database available at https://lomanlab.github.io/mockcommunity/mc_databases.html. The eukaryotic taxonomies were further assigned using the PhyloDB and MMETSP databases associated with EUKulele (commit# fb8726a; available at https://github.com/AlexanderLabWHOI/EUKulele). Subsequently, contigs binned in eukaryotic MAGs were independently validated using WHOkaryote (63), which distinguishes eukaryotic and prokaryotic contigs in metagenomes based on gene structure. Consensus taxonomy per contig was then used for downstream analyses and association with ARGs.

### Identification of antimicrobial resistance genes, antibiotic biosynthesis pathways, and BGCs.

For the prediction of ARGs, the IMP-generated contigs were used as input for PathoFact (64). Identified ARGs were further collapsed into their respective AMR categories in accordance with the Comprehensive Antibiotic Resistance Database (CARD) (65). PathoFact utilizes two distinct tools: DeepARG-LS, a deep-learning model, and RGI, based on homology and SNP models, to identify ARGs, therefore possibly also detecting resistance genes within eukaryotic genomic fragments. Subsequently, the raw read counts per open reading frame (ORF), obtained from PathoFact, were determined using FeatureCounts (66).

To identify pathways for the biosynthesis of antibiotics, we assigned KEGG orthology (KOs) identifiers to the ORFs using a hidden Markov model (HMM) (67) approach using *hmmsearch* from HMMER 3.1 (68) with a minimum bit score of 40. Additionally, we linked the identified KOs to their corresponding KEGG orthology pathways and extracted the pathways annotated as antibiotic biosynthesis pathways by KEGG. Both the identified ARGs and KEGG pathways were then further linked to associated bacterial taxonomies using the contig classification. We further identified BGCs within the MAGs using antiSMASH (ANTIbiotics & Secondary Metabolite Analysis SHell) (29) and annotated these using deepBGC (30). Owing to the possibility that a single tool such as deepBGC may not be ideal for identifying BGCs, we additionally used GECCO to (69) identify and annotate BGCs. Since GECCO was trained on the MiBiG database (70), we expected to improve our detection sensitivity with the additional approach. BGCs that were identified by both tools, including those unique to each, were used for downstream analyses. To link BGCs and ARGs, we linked the resistance genes to the assembled contigs, followed by identifying the corresponding bins (MAGs) to which said contigs belonged. We subsequently extracted the genomic coordinates from the individual GenBank (.gbk) files for each of the BGCs, based on the deepBGC and GECCO analyses. The genomic coordinates were used to establish the location of the BGCs with respect to the ARGs identified via PathoFact. For the analyses described above, we only considered ARGs and BGCs within a maximum distance of 2 genes from each other, which corresponded to up to 2.5 Kb. This was based on a previous report by Conway et al. (71), who reported that, on average, there are 1.98 genes in a bacterial operon.

### Validation and phylogenetic analyses of putative ARGs.

To ensure the accuracy of the ARGs identified within both pro- and eukaryotes and assess their homology to sequences within existing databases, the respective ARG sequences were retrieved and the following analyses performed. The retrieved sequences were cross-referenced using DIAMOND (72) against the UniProtKB database (73) to determine if the putative ARGs found within pro- and eukaryotes encoded other functions. To further determine how the putative ARGs from GFSs relate to their homologs, the existing ARG database (i.e., CARD), a phylogenetic analysis was performed. For the phylogenetics analyses, all ARG nucleotide sequences were collected from the CARD database and grouped by classification. Pro- and eukaryotic ARGs from the GFS samples with similar classifications were collated respectively, into multifasta files along with the reference sequences, and a multiple sequence alignment was performed using MAFFT (74) with default settings. The aligned sequences were subsequently trimmed with trimAl (75) using “*-gt 0.9*” as a threshold for gap filtering. Lastly, IQ-TREE 2 (76) was used to construct a phylogenetic tree using trimmed multiple sequence alignment with at least 10 FASTA sequences. The built trees were visualized using the *ggtree* package (77) using version 3.6 of the R statistical software package (78).

### Data analysis.

The relative abundance of the ORFs was calculated based on the RNum_Gi method described by Hu et al. (79). Figures for the study, including visualizations derived from the taxonomic and functional analyses, were created using the R statistical software and the *tidyverse* package (80). Alluvial plots were generated using the *ggalluvial* package (81), while heatmaps were generated using the *ComplexHeatmap* package (82) developed for R. The corresponding visualization and analysis code is available at https://github.com/susheelbhanu/rock_biofilm_amr.

### Data availability.

The Biosample accession IDs listed under Table S4 can be found on NCBI under the BioProject accession number PRJNA781406. The analysis code for IMP and downstream analys is detailed at https://git-r3lab.uni.lu/susheel.busi/nomis_pipeline. Binning and manual refinement of eukaryotic MAGs was done as described here: https://git-r3lab.uni.lu/susheel.busi/nomis_pipeline/-/blob/master/workflow/notes/MiscEUKMAGs.md. All visualization and analysis code is available at https://git-r3lab.uni.lu/susheel.busi/rock_amr and https://github.com/susheelbhanu/rock_biofilm_amr.
