# Supplementary material for: Glacier-Fed Stream Biofilms Harbor Diverse Resistomes and Biosynthetic Gene Clusters
Source: Microbiol Spectr. 2023 Jan 23;11(1):e04069-22. doi: 10.1128/spectrum.04069-22 (PMC9927545; doi:10.1128/spectrum.04069-22)

Glacier-fed stream biofilms harbour diverse resistomes and biosynthetic gene clusters

Susheel Bhanu Busi[1,‡,*], Laura de Nies[1,‡], Paraskevi Pramateftaki[2], Massimo Bourquin[2], Tyler J. Kohler[2], Leïla Ezzat[2], Stilianos Fodelianakis[2], Grégoire Michoud[2], Hannes Peter[2], Michail Styllas[2], Matteo Tolosano[2], Vincent De Staercke[2], Martina Schön[2], Valentina Galata[1], Paul Wilmes[1,*,#] and Tom Battin[2,#]

Supplementary Legends

Supplementary figure 1. Ordination analyses reveal the (dis)similarity of the GFS resistomes

(a) Principal component analyses depicting the overall similarity of the individual GFS resistomes. Each dot represents the resistome predicted from a single metagenome. SA: Southern Alps. CU: Caucasus. (b) Biplot demonstrating the underlying factors, i.e. ARG abundances across 29 AMR categories, driving the similarity within the GFS epilithic resistomes.

Supplementary figure 2. Bacterial and eukaryotic phyla encode AMR

(a) Relative abundance of the bacteria associated with AMR. The stacked bar plots are facetted by the individual GFSs where the epilithic biofilms were collected. The colors represent the individual phyla. (b) Stacked bar plots indicating the relative abundance of the AMR encoded by eukaryotes.

Supplementary figure 3. Phylogenetic analyses of prokaryotic ARGs

(a) Phylogenetic tree generated using the ARG sequences from the reference database, i.e. CARD, with the TETA (tetracycline) resistance gene sequence retrieved from GFS prokaryotes. (b) Tree built using the ERM (MLS phenotype) resistance genes retrieved from the prokaryotes and CARD databases. Group in both figures indicates the origins of the sequence, i.e. CARD database or prokaryote.

Supplementary figure 4. Sequence homology to UniProtKB database

(a) Bar plot depicting the number of identified hits comparing the pro- and eukaryote-derived ARGs against the UniProtKB database. total: total number of input sequences used for the DIAMOND analysis; hits: number of matches found within UniProtKB; greater_than_50: number of hits with more than 50% identity; total_percent: percentage of hits to UniProtKB, estimated based on total input sequences; greater_50_percent: percentage of hits with more than 50% identity. (b) BLAST output from UniProtKB for a consensus TETA sequence from eukaryotic ARGs.

Supplementary figure 5. Antibiotic synthesis pathway assessment via KEGG orthology

(a) Relative abundance of KEGG pathways associated with antibiotic synthesis across the 21 epilithic biofilms. (b) Bar plots indicating the relative abundance of the antibiotic associated KEGG pathways mediated by bacteria and eukaryotes. (c) Normalised relative abundance of pathways associated with antibiotic production in the KEGG database, juxtaposed with the various phyla encoding these genes.

Supplementary data

Supplementary table 1. Sample metadata

Supplementary table 2. List of ARGs identified across 21 GFS epilithic biofilms

Supplementary table 3. Pro- and eukaryotic ARG comparison with UniProtKB

Supplementary table 4. List of BGCs across all MAGs

Supplementary table 5. NCBI accession metadata

Supplementary Figure 1

a

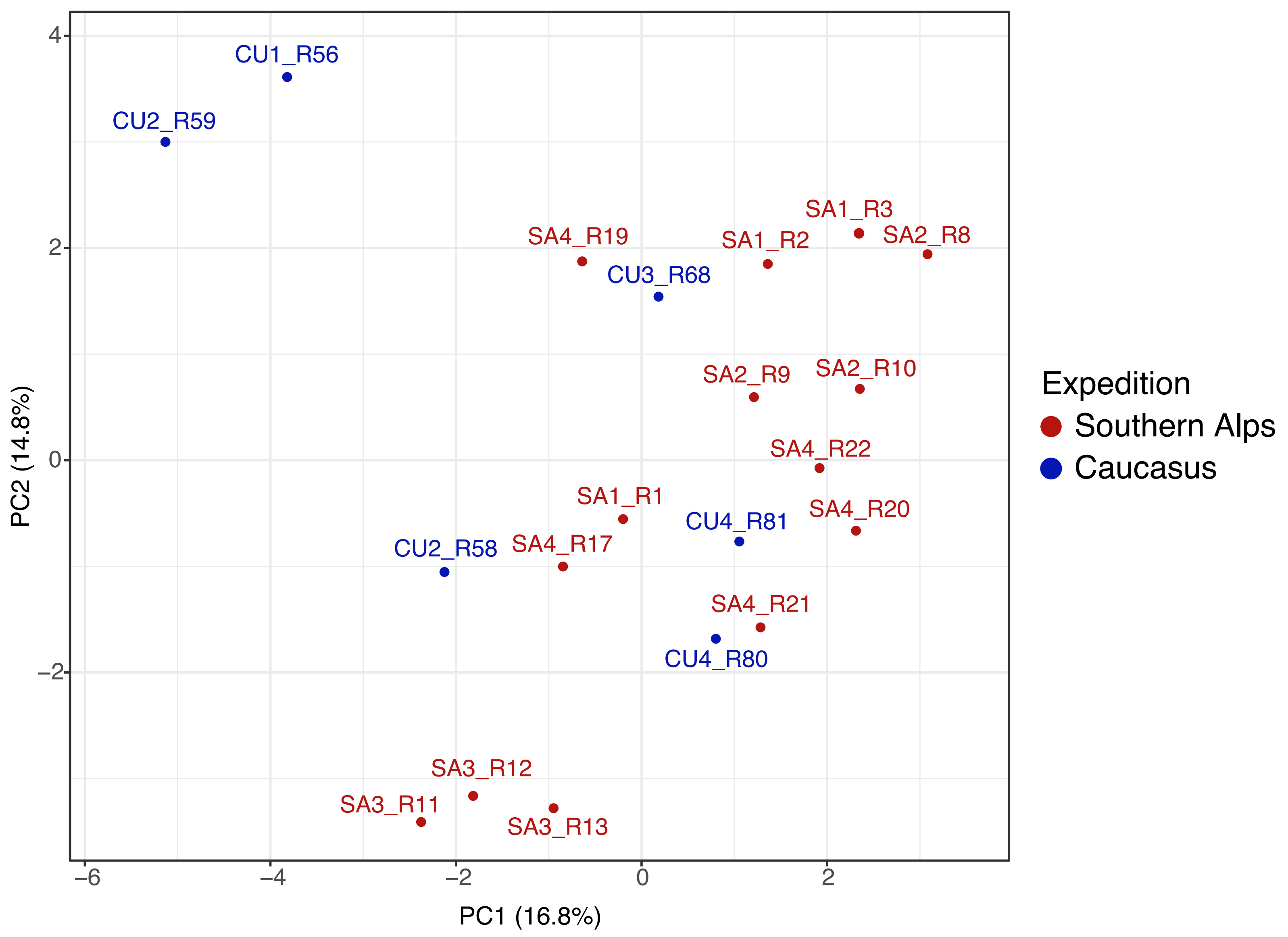

b

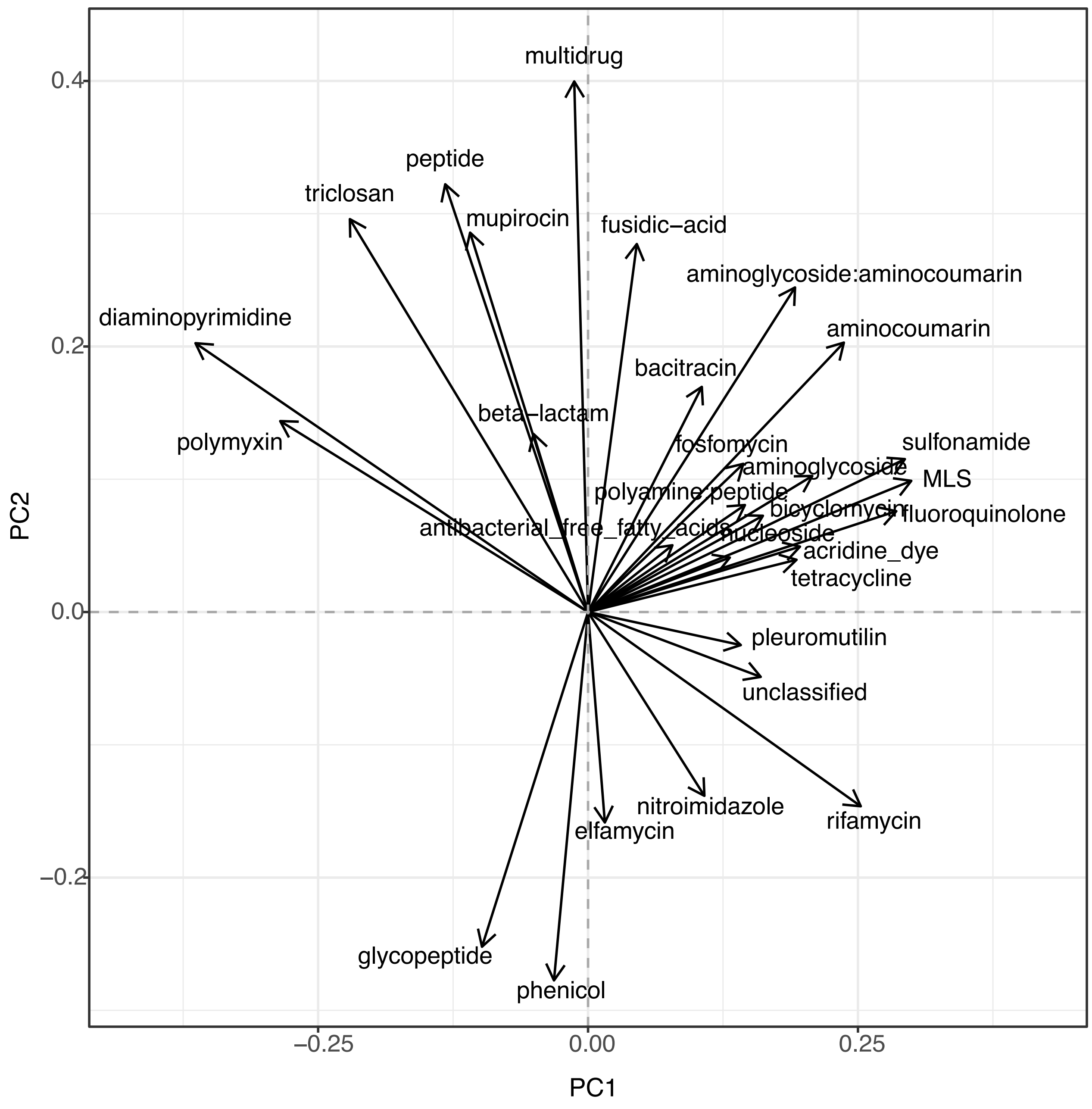

Supplementary Figure 2

a

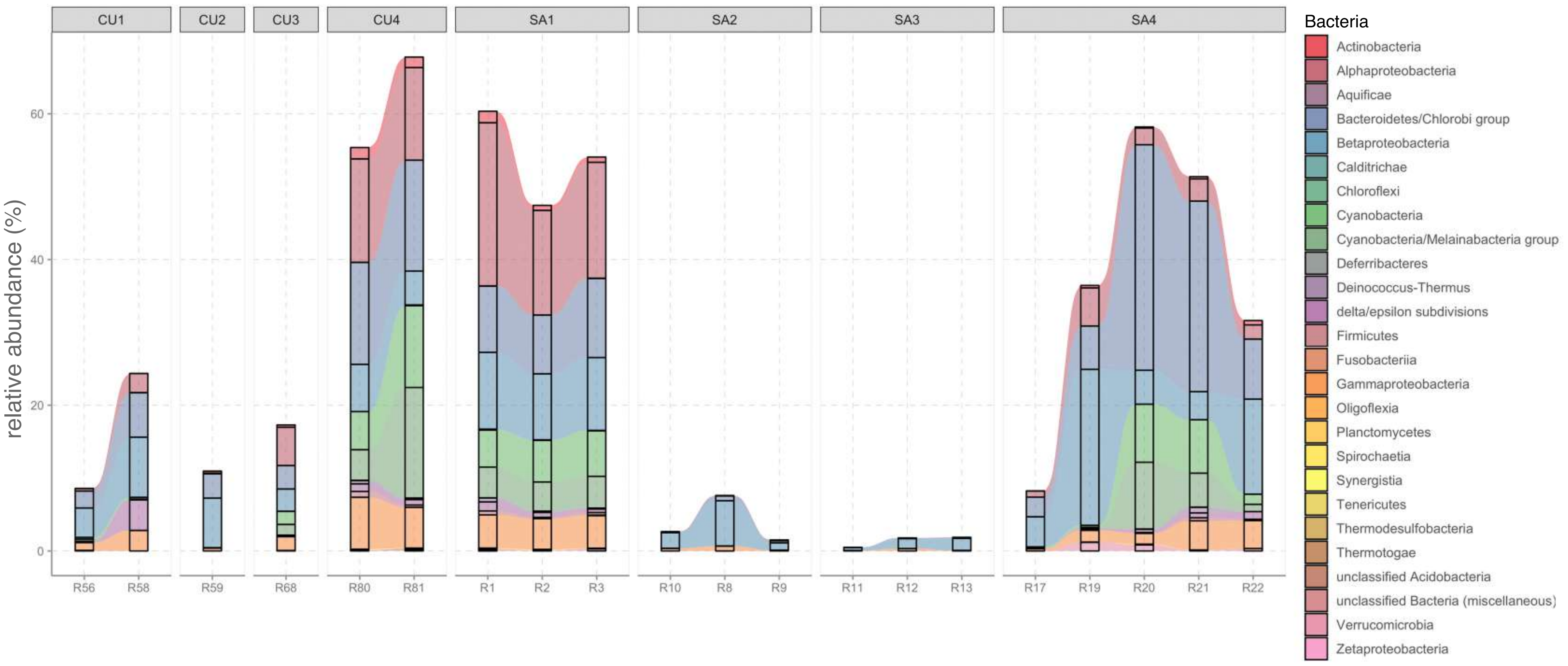

b

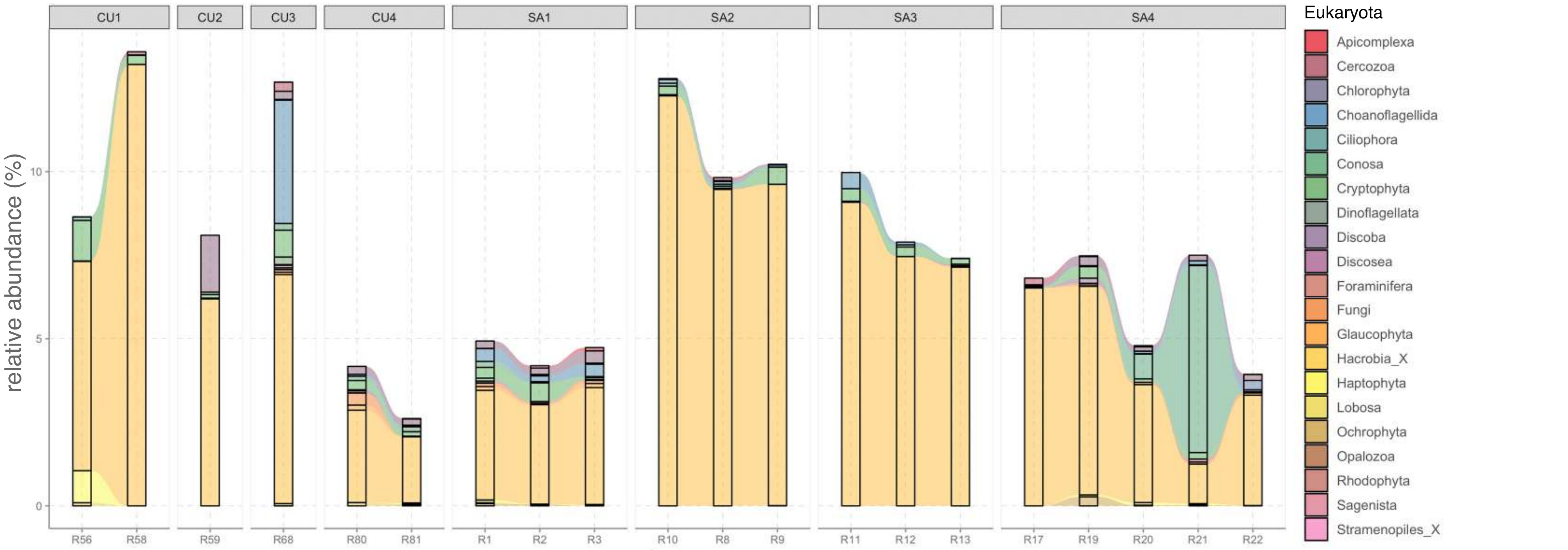

Supplementary Figure 3

a

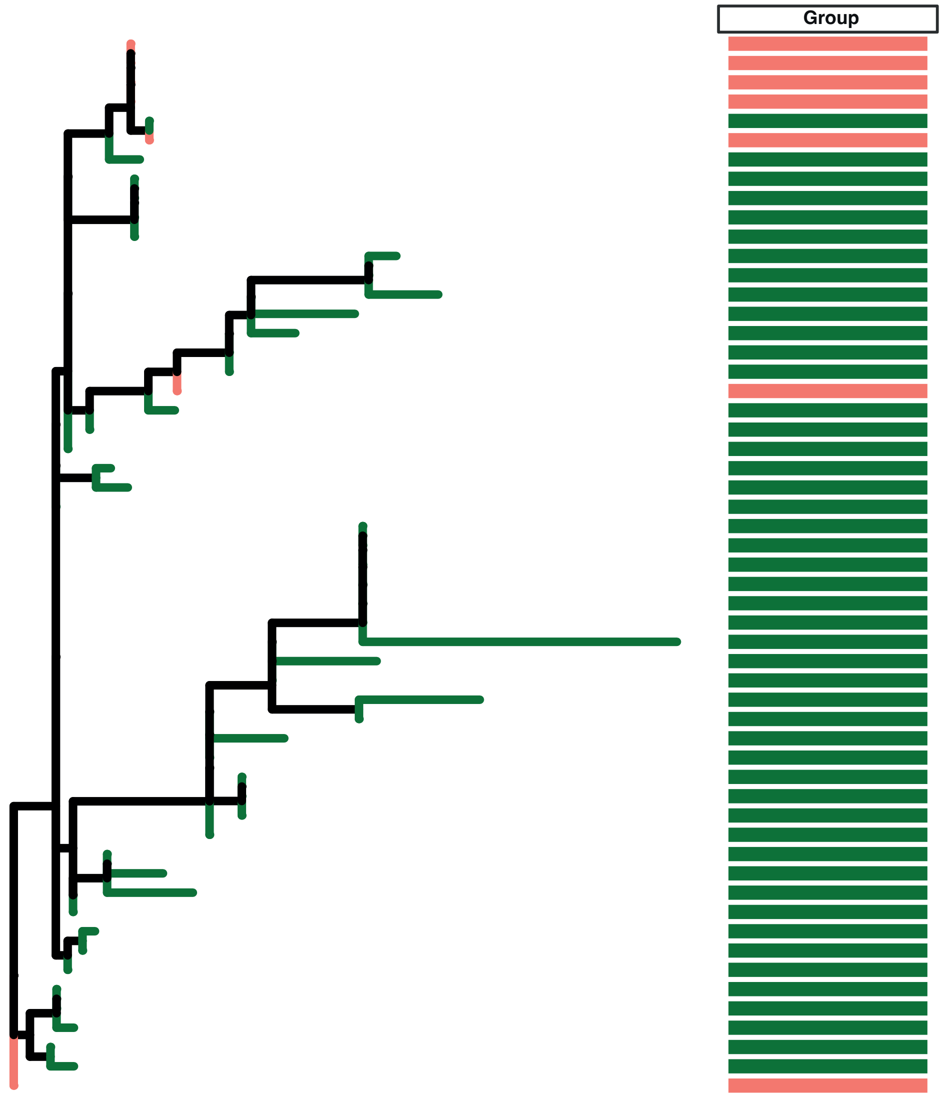

b

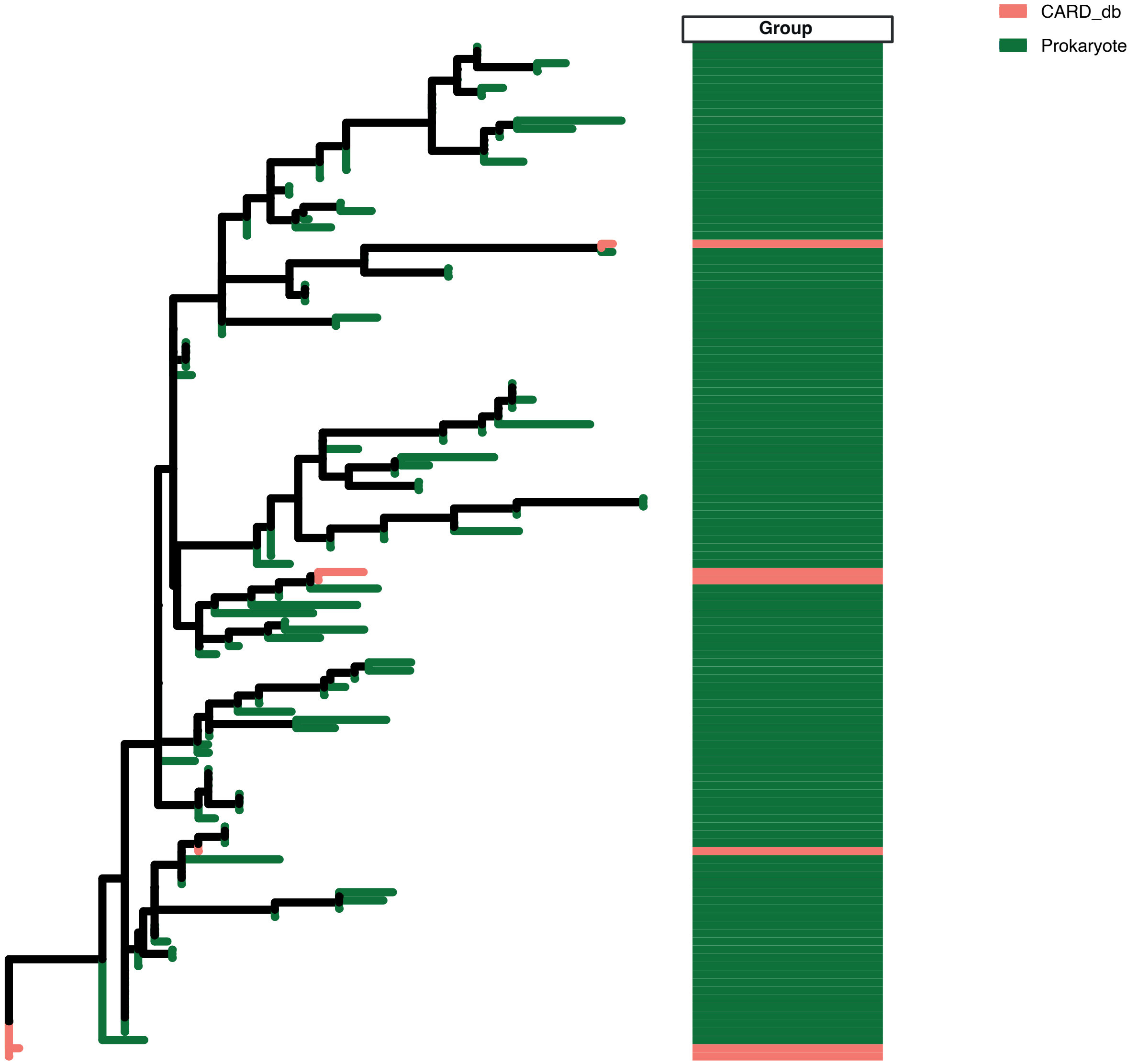

Supplementary Figure 4

a

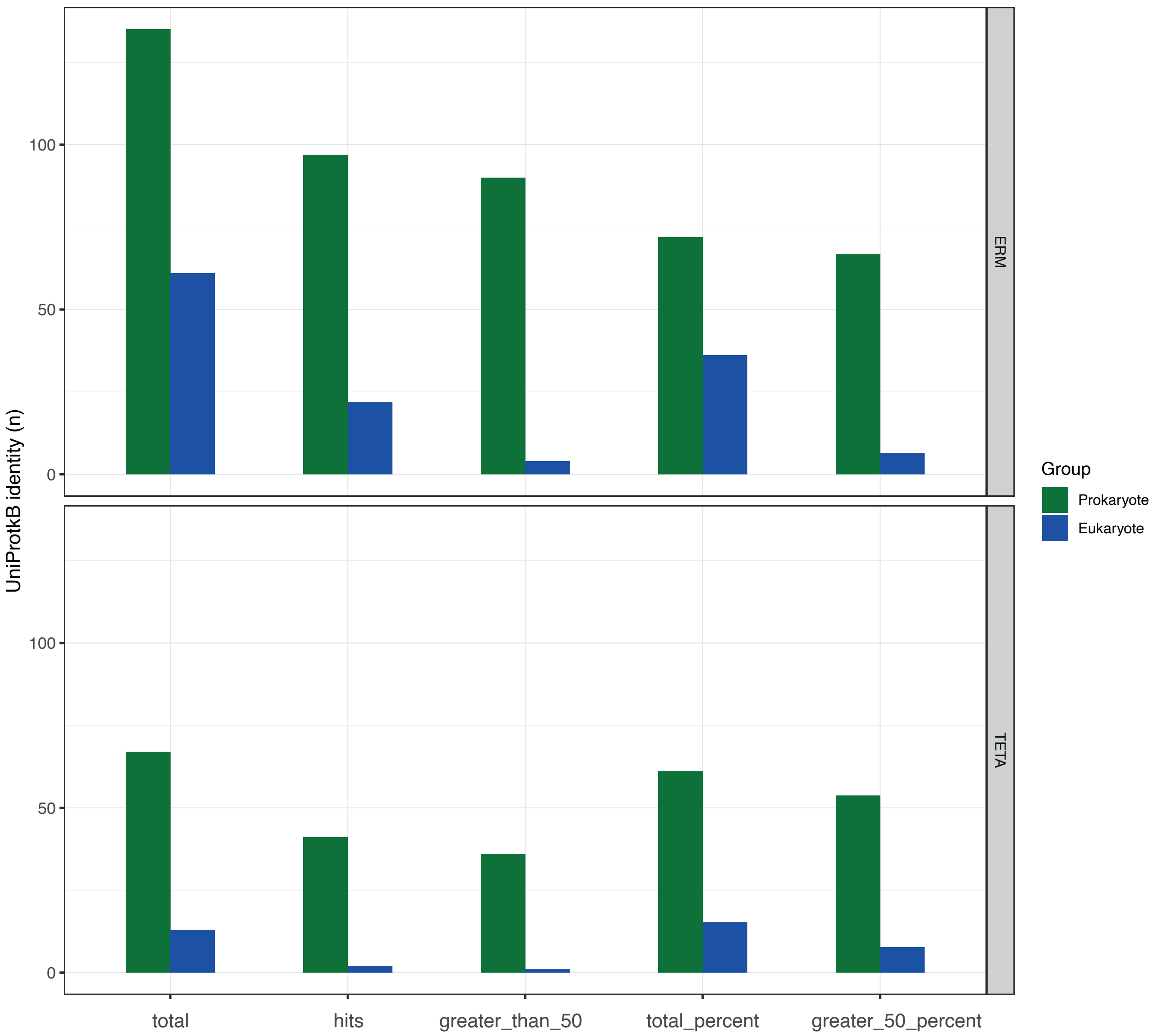

b

Entry	Protein names	Match hit (100 80 60)	Identity
A0A4P1RVP7	Uncharacterized protein (Lupinus angustifolius)		35.2%
A0A1J1GKB4	Uncharacterized protein (Plasmodium relictum)		27.5%
A0A2J6MNT3	tRNA 2-selenouridine(34) synthase MnmH (Burkholderia sp. WAC0059)		32.6%
R6WXQ2	Putative phage virion morphogenesis protein (Prevotella sp. CAG:474)		30.7%
A0A553ND90	**SLBP_RNA_bind domain-containing prote** Putative phage virion morphogenesis protein		28.6%
A0A1C0VT37	Peptidase M61 (Oscillatoriales cyanobacteri...)		33.3%
A0A812BMZ4	Hypothetical protein (Sepia pharaonis)		57.6%
A0A811KQQ8	Hypothetical protein (Bursaphelenchus okinawaensis)		40.0%
A0A4Q9FRU3	Endo-1,4-beta-xylanase (Hyunsoonleella pacifica)		31.7%
A0A073CD96	**PDZ domain-containing protein** (Planktothrix agardhii NIVA-C...)		26.7%
A0A498HVS2	Uncharacterized protein (Malus domestica)		39.1%

Supplementary Figure 5

a

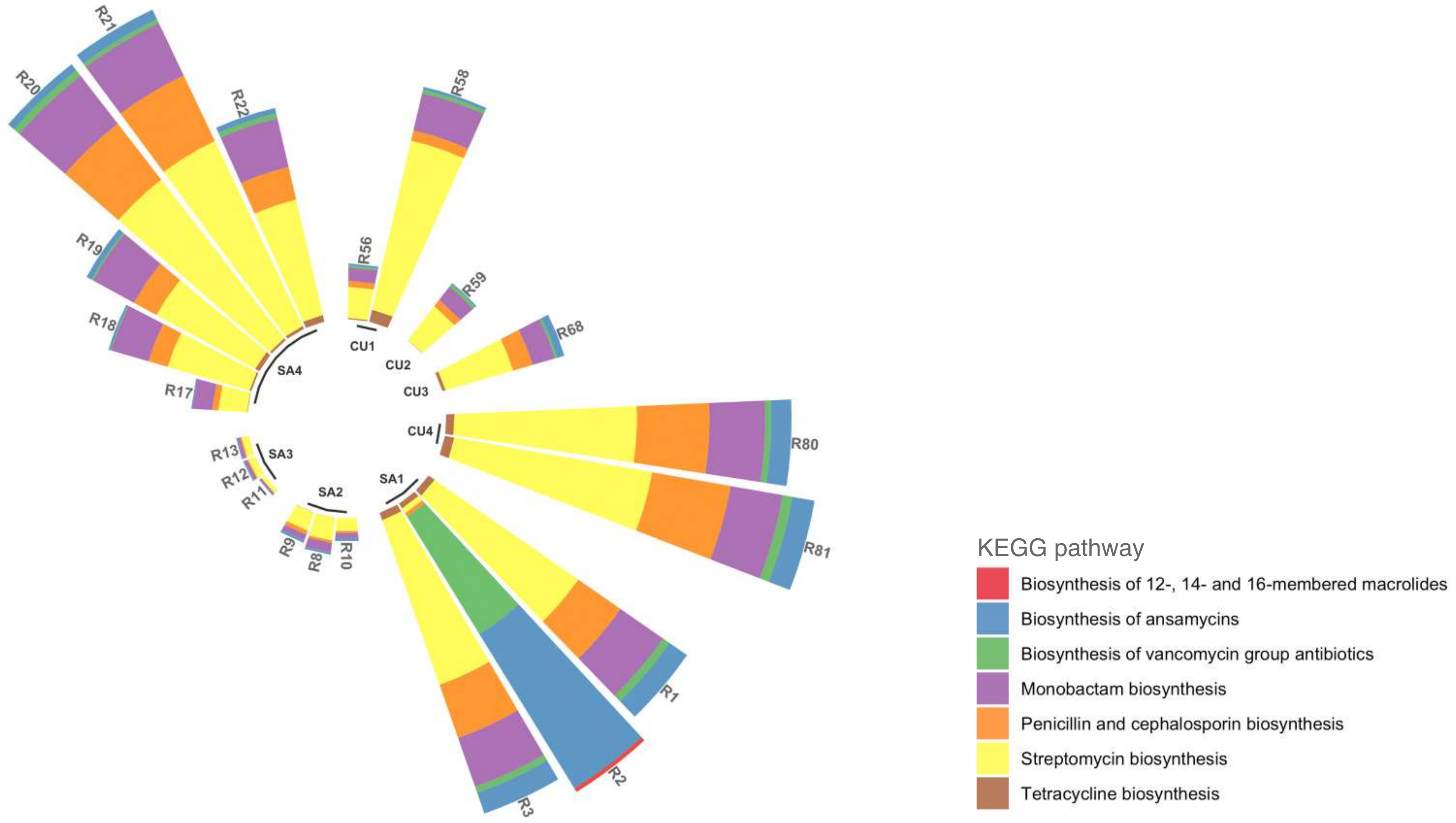

b

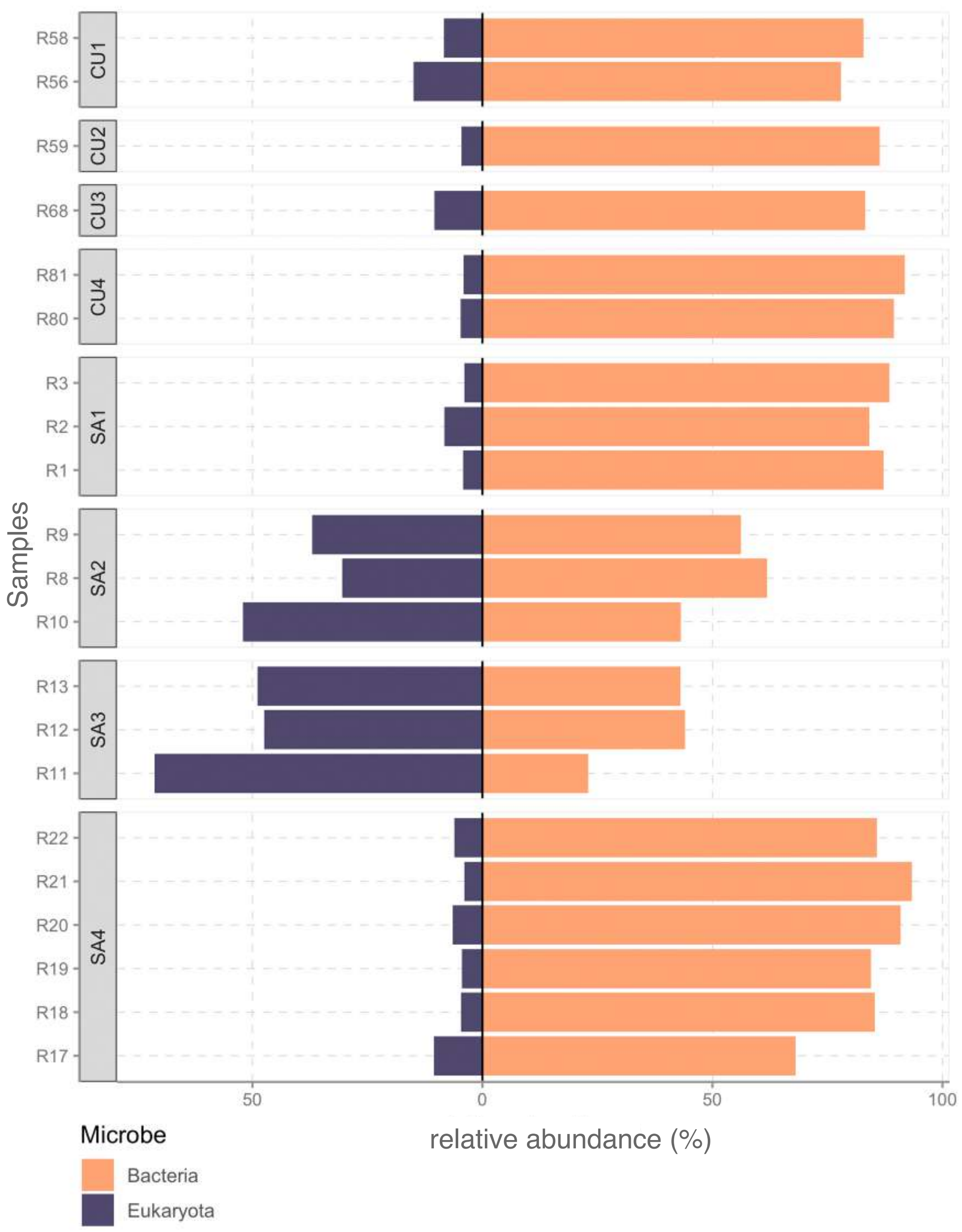

c

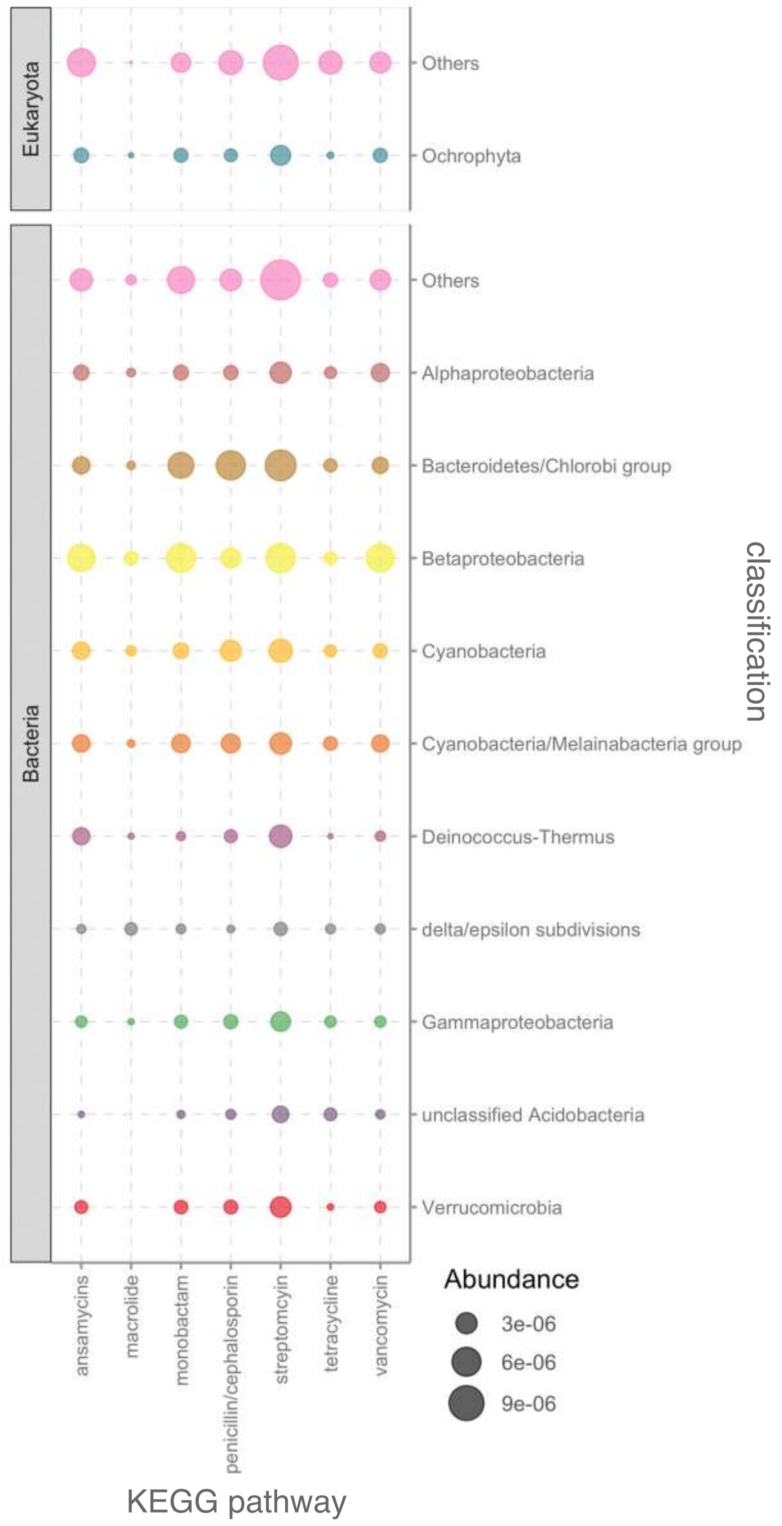

Supplement: Supplemental file 6 — Supplemental material. Download spectrum.04069-22-s0001.pdf, PDF file, 1.3 MB [file spectrum.04069-22-s0001.pdf]
